# No Relationships Between the Within-Subjects’ Variability of Pain Intensity Reports and Variability of Other Bodily Sensations Reports

**DOI:** 10.3389/fnins.2019.00774

**Published:** 2019-08-13

**Authors:** Mariana Agostinho, Rita Canaipa, Liat Honigman, Roi Treister

**Affiliations:** ^1^CIIS, Centre for Interdisciplinary Health Research, Institute of Health Sciences, Catholic University of Portugal, Lisbon, Portugal; ^2^The Clinical Pain Innovation Lab, Faculty of Social Welfare and Health Sciences, University of Haifa, Haifa, Israel

**Keywords:** pain assessment, pain intensity, interoceptive awareness, subjective measures, within-subject variability

## Abstract

**Purpose:**

The subjective nature of pain assessment and its large variance negatively affect patient–health care provider communication and reduce the assay sensitivity of pain clinical trials. Given the lack of an objective gold standard measure, identifying the source (true or error) of the within-subject variability of pain reports is a challenge. By assessing the within-subjects variability of pain and taste reports, alongside with interoceptive measures, the current study is aimed to investigate if the ability to reliably report bodily sensations is a cross-modal characteristic.

**Patients and Methods:**

This prospective study enrolled healthy volunteers from local universities. After consenting, subjects underwent the Focus Analgesia Selection Task (FAST), to assess within-subjects variability of pain reports in response to experimental noxious stimuli; a taste task, which similarly assesses within-subjects variability of tastes (salty and sweet) intensity reports; and the heartbeat perception task, an interoceptive task aimed to assess how accurate subjects are in monitoring and reporting their own heartbeat. In addition, all subjects completed the Multidimensional Assessment of Interoceptive Awareness (MAIA), the Perceived Stress Scale (PSS), and Hospital Anxiety and Depression Scale (HADS). Spearman’s correlations were used to assess relations between all measures.

**Results:**

Sixty healthy volunteers were recruited. Variability of intensity reports of different modalities were independent of each other (*P* > 0.05 for all correlations). The only correlation found was within modality, between variability of intensity reports of salt and sweet tastes (Spearman’s *r* = 0.477, *P* < 0.001). No correlations were found between any of the task results and questionnaire results.

**Conclusion:**

Within-subjects variability of pain reports do not relate to variability of reports of other modalities or to interoceptive awareness. Further research is ongoing to investigate the clinical relevance of within-subjects’ variability of pain reports.

## Introduction

The assessment of pain intensity, such as intensities of other subjective experiences, is challenging. Both clinical care and research findings depend on such subjective self-reports, reported on one-dimensional scales that are variably understood by subjects (Dionne et al., 2005). Such limitations are reflected in experts’ concerns regarding the appropriateness of pain intensity measures as primary outcomes in chronic pain populations (Ballantyne and Sullivan, 2015), and in lack of use of such scales by pain clinicians (Bačkonja and Farrar, 2015).

Patients vary in the tendency to demonstrate within-subjects variability of pain reports. Previous research demonstrated that within-patients fluctuations (over days) of clinical pain relates to patients’ response to treatment (Harris et al., 2005; Farrar et al., 2014). Within-subjects variability of pain reports can also be assessed by the Focused Analgesia Selection Test (FAST) task (Treister et al., 2017), which is based on exposing subjects to repeated noxious stimuli of various intensities in a blinded manner.

Assessing the relations between stimulus intensities and pain reports, as done in the FAST procedure, allows assessing the variance in response to the same and to other (lower or higher) stimuli intensities. The FAST was designed to minimize peripheral modulatory effects (i.e., habituation and sensitization) that could account for true variability. Hence, the within-subjects variability measured in the FAST reflects (at least partly) subjects’ ability to reliably report pain. As shown, patients’ performance in the FAST are clinically relevant: the latter were associated with patients’ ability to report changes in clinical pain (Treister et al., 2017) and with the day-to-day variability of clinical pain (Treister et al., 2019). However, it is not clear if the tendency of a subject to reliably report pain is a pain-specific, or if it is a general, cross-modal characteristic. To shed more light on this open question, for the purpose of this investigation, we modified a taste perception task, to allow the assessment of within-subject variability of taste intensity reports (similarly the FAST task), from which subjects’ ability to reliably report intensity of tastes could be inferred.

Relations between sensitivity to pain and other non-painful sensations were investigated in the past. As such, in Hummel et al. (2011), the relations between responses to two noxious stimuli modalities (intranasal gaseous CO_2_ and cutaneous electrical stimuli) and non-noxious stimuli modalities (taste and smell) were assessed. No cross-modal associations were found, implying that sensitivity cannot be generalized across senses. However, unlike in the Hummel et al. (2011) study, in which sensitivity to stimuli of various intensities was assessed, the focus of our study is to assess if the ability to reliably report pain and taste is related.

In contrast to the assessment of within-subject variability of pain reports, the assessment of reporting accuracy of other bodily sensations, termed Interoception, has been intensively investigated. Previously seen merely as the sense of visceral sensations, interoception is currently defined as “the sense of the physiological condition of the entire body” (Craig, 2014; Ceunen et al., 2016). Interoceptive signals ascend from the periphery in two main pathways (Craig, 2009), the spinothalamic and lemniscal tracts, which are integrated at multiple levels, among which the medial and the anterior insular cortex play a primary role (Engström et al., 2015). It is often linked to pain and temperature, cardiorespiratory function, hunger, thirst, stress, vasomotor flush and respiration, and emotion experience (Wiens et al., 2000). Interoception has been assessed using several methods and modalities. A common method is based on sensitivity to detect one’s own heartbeats, termed the heartbeat detection task (Schandry, 1981). Other methods and questionnaires have been used to assess different interoception modalities, such as gastric function (Whitehead and Drescher, 1980; Hobday et al., 2001; Chen et al., 2005; Herbert et al., 2012), respiratory function (Harver et al., 1993; Webster and Colrain, 2000; Daubenmier et al., 2013; Faull et al., 2016; Zechman et al., 2017), tactile acuity (Van Boven and Johnson, 1994; zu Eulenburg et al., 2013), and taste (Whitehead et al., 1977; Harver et al., 1993; Flor et al., 1999; Herbert and Pollatos, 2012; Mehling et al., 2012; Michael et al., 2015; Garfinkel et al., 2016).

Despite theoretical suggestions of multiple integrations of the interoceptive information (Craig, 2014; Smith and Lane, 2015), it is unknown whether there is a general interoceptive ability. Based on the comparison between modalities, only three studies found moderate associations between modalities. Two between heartbeat perception task and gastric perception (Whitehead and Drescher, 1980; Herbert et al., 2012) and another between the heartbeat task and the perception of skin conductance (Steptoe and Noll, 1997). Several other studies assessing perception of multiple interoception modalities, such as detection of heartbeat, gastric, and respiratory perception, did not find correlations between different modalities (Harver et al., 1993; Vaitl, 1996; Werner et al., 2009; Garfinkel et al., 2016). A recently published study directly investigated the relations between six modalities of interoception (Ferentzi et al., 2018). The results indicated lack of correlations between different modalities.

Most of the tasks used in these previous studies assessed the sensitivity of subjects in response to stimuli of each modality; however, the question whether the ability to reliably report bodily sensations is a general trait was never investigated before. The current study aim was to assess relations between the tendency of subjects to reliably report pain and taste intensities (reflected by low within-subject variability of pain and taste reports during the experimental tasks) with subjects’ interoceptive ability, assessed by both the heartbeat detection task and the Multidimensional Assessment of Interoceptive Awareness (MAIA) questionnaire.

## Materials and Methods

### Subjects

The study sample included healthy volunteers recruited from local universities of Lisbon. Experiments were conducted in accordance with the Declaration of Helsinki and with the approval of the Ethical Board of the Institute of Health Sciences, Catholic University of Portugal (Protocol no. 31 de April 24th, 2017). Written informed consent was obtained from each participant before the beginning of the experiment, and afterward, a code number was attributed. Participants enrolled into the study only if they met the following criteria: (1) age above 18; (2) absence of acute or chronic pain disorders; (3) no reports of psychiatric, cognitive, and/or neurological disorders; and (4) no chronic use of medications except for oral contraceptives. Participants were excluded if (1) they were pregnant or breastfeeding, (2) they had any persistent or severe infection within 30 days of baseline, (3) they had formal diagnosis of any uncontrolled medical condition, and (4) they were unable to provide informed consent, communicate, and understand the purpose and the instructions of the study.

Power calculation was performed using G^∗^Power (version 3.1.9.4). Based on an alpha of 0.05 and a power of 0.8, 64 subjects are needed to detect significant correlation of medium effect size (0.3).

### Instruments and Procedures

#### The Focused Analgesia Selection Test

Focus Analgesia Selection Task was developed to assess pain-reporting skills in response to repeated administration of thermal noxious stimuli of varying intensities applied to the ventral surface of the subject’s non-dominant arm. It uses the Medoc^®^ Thermal Sensory Analyzer II incorporating a Peltier element-based thermode (30 mm^2^ × 30 mm^2^). Subjects are instructed to verbally rate their perceived pain intensity in response to each stimulus on a 0–100 numerical rating scale (NRS), ranging from 0, denoting “no pain,” to 100, denoting “the worst imaginable pain.” The temperature increased from a baseline of 32°C, peaking for 3 s at one out of seven predetermined temperatures (44°C, 45°C, 46°C, 47°C, 48°C, 49°C, or 50°C) and then decreasing down to baseline, with a total stimulus duration (including ramping up and down) of 8 s. Each temperature is presented seven times in a random block-ordered design (total of 49 stimuli), based on a previously described protocol (Treister et al., 2017). The location of the thermode was adjusted every 10 stimuli to minimize sensitization and/or habituation effects with inter-stimulus intervals (ISIs) of 15 s. The FAST procedure total duration is about 20 min.

Pain scores captured during the FAST allow calculating the following three FAST outcomes, each capturing a different aspect of the within-subject variability, and reflecting the ability of subjects to reliably report pain. (1) *R*^2^ is calculated by using a power model regression. Disparity between the predicted function and actual scores could be a result of inaccuracy or unreliability. Close concordance between actual and predicted scores are expressed by higher *R*^2^, and suggests greater accuracy and reliability. (2) ICC is computed using a two-way mixed model for the seven presentations of each of the seven stimuli intensities. An ICC score approaching 1 denotes a high degree of reliability (or the agreement in responses to the same stimulus over several presentations). (3) The CoV is the ratio of the standard deviation (SD) to the mean, calculated separately for each stimulus intensity. The mean CoV of the seven CoVs (of each stimulus intensity) is calculated. A higher CoV demonstrates a larger variability, or lower reliability of pain reports.

#### Taste Perception Task

This task is a modification of Hendi and Leshem (2014) procedure, which was originally developed to assess the sensitivity to salty and sweet tastes. Unlike the original purpose of the procedure, in this study, the procedure was modified to allow the assessment of within-subject variability of taste intensity reports. Participants were asked to avoid eating, drinking (except water), and smoking 2 h before the test. During the task, the experimenter sprayed each solution into the participant’s oral cavity. The volume of each spray was 0.29 cc (Leshem, 2017), and subjects were requested to wash their oral cavity with fresh mineral water between each application of the spray. Subjects randomly began the taste task with either salty or sweet taste series, followed by the other taste. Each concentration was repeated five times (a total of 25 repeats for each taste), in a randomized order (excluding sequential concentrations). Participants were asked to indicate how strong was the taste, for each concentration of taste, on an NRS ranging from 0 “not feeling” to 100 “most strong.” As in the FAST procedure, the taste procedure outcomes are the *R*^2^, ICC, and CoV, calculated in the same manner as in the FAST, reflecting subjects’ ability to reliably report taste intensity reports. Concentrations (from low to high) of the salt solutions were 0.09, 0.28, 0.85, 1.71, and 2.56 M. Concentrations of the sweet solutions were 0.03, 0.09, 0.26, 0.39, and 0.79 M. These concentrations were chosen based on pilot studies, in which the appropriate concentrations were identified, to minimize floor or ceiling effects, and to produce significantly different perceived intensities between concentrations.

#### Heartbeat Perception Task

The heartbeat perception task assesses the individuals’ accuracy of subjective heartbeat reports. This task, named the Mental Tracking Method, was developed by Schandry (1981) to assess interoception accuracy using three heartbeat counting phases with varying length. First, participants fitted to physiological recording equipment to assess true heartbeat through electrocardiography (BITalino device, Plux Wireless Biosignals, SA, Lisbon, Portugal). The experimental task consisted of a 5-min resting period to assess baseline measures. Then, when a voice signal was presented by a research assistant, the subject is asked to pay attention and count his/her heartbeats silently, focusing only on bodily feelings. Next, after offset voice signal was given, the subject is asked to report the number of counted heartbeats. The following instructions are given: “Without manually checking, count silently each heartbeat you feel in your body from the time you hear ‘start’ to when you hear ‘stop’.” Subjects were instructed to avoid any kind of physical manipulation (pressure points and respiratory manipulation) that might ease detection. The task was performed three times with varying length (25, 35, and 45 s) in the following order: rest (60 s)–perception (25 s)–rest (30 s)–perception (35 s)–rest (30 s)–perception (45 s)–rest (60 s). The subject was unaware to the different length of each round. Heart rate was assessed using Ag/AgCl electrodes per Eithovens’ triangle, connected to the BITalino device. Heartbeat perception accuracy was calculated, for each subject, as an error score between counted heartbeats reported and actual heartbeats obtained by ECG, according to the formula: Interoception Accuracy Score:

$$\begin{array}{c} =\frac{1}{3}\sum [1-\left(\mathrm{recorded heartbeats}-\mathrm{counted heartbeats}\right)\\ \;/\mathrm{recorded}\mathrm{heartbeats}] \end{array}$$

Interoception accuracy scores vary between 0 and 1. Higher scores indicate better interoception accuracy.

### Questionnaires

#### Sociodemographic Questionnaire

Participants indicated their age, sex, height and weight, health condition, medication (last 48 h, contraceptives), last menstruation, and consumption habits (alcohol, tobacco, and drugs).

In addition, the following Patients Reported Outcome measures, which are associated with pain or interoception, were assessed.

#### Perceived Stress Scale

Perceived Stress Scale (PSS) is a brief instrument, used in community samples to assess to what degree situations in participants’ life were appraised as stressful (Cohen et al., 1983). In response to each item, participants report their feelings on a five-point Likert scale during last month. The validated Portuguese version of this instrument was considered adequate and was used (Moreira, 2002).

#### Hospital Anxiety and Depression Scale

The Hospital Anxiety and Depression Scale (HADS) is a brief instrument commonly used to assess anxiety and depression in a non-psychiatric population (Zigmond and Snaith, 1983). It consists of 14 items (response scale 0–3) that are divided into two subscales measuring either anxiety or depression feelings during the past week. The validated Portuguese version was used (McIntyre et al., 1999). The results vary from 0 to 21, with higher scores indicating higher levels of depression or anxiety. The severity of anxiety and depression is classified as follows: 0–7 = normal, 8–10 = light, 11–14 = mild, and 15–21 = severe.

#### Multidimensional Assessment of Interoceptive Awareness

Interoceptive awareness (IAw) was assessed by the Portuguese version (Machorrinho, 2017) of the original English-language MAIA (Mehling et al., 2012). The MAIA is composed of 33 items scored on a six-point Likert scale. This multidimensional instrument measures IAw on seven subscales: (1) Noticing, the awareness of one’s body sensations (three items); (2) Not-distracting, the tendency not to ignore or distract oneself from sensations of pain or discomfort (four items); (3) Not-worrying, the tendency not to experience emotional distress or worry with sensations of pain or discomfort (four items); (4) Attention regulation, the ability to sustain and control attention to body sensation (seven items); (5) Emotional awareness, the awareness of the connection between body sensations and emotional states (five items); (6) Self-regulation, the ability to regulate psychological distress by attention to body sensations (seven items); (7) Trusting: the experience of one’s body as safe and trustworthy (three items). The score for each scale is calculated by averaging the scores of individual items, and thus can range 0–5.

### Experimental Protocol

The experiments were conducted at NeuroSer Clinic and university laboratory. At the beginning of the experimental session, all subjects underwent short training in order to familiarize them with the devices, the sensations evoked by the painful stimulation, and the rating task. After the familiarization stage, the experiment begun with the FAST procedure. Thereafter, all subjects performed the heartbeat task, which began with 5-min baseline measures recordings, followed by a 10-s familiarization phase. Upon completion of the heartbeat detection task, the taste procedure was familiarized and preformed. The total duration of the experimental session was approximately 1.5 h, and participants were rewarded for their participation with credits for neuroscience subject.

### Statistical Analyses

Data were processed and analyzed using Excel (Microsoft Corp., Redmond, WA, United States) and SPSS software version 23 (SPSS, Inc., Chicago, IL, United States). Descriptive statistics were used to present demographic and baseline characteristics.

As most variables were non-normally distributed (tested by Kolmogorov–Smirnov and Shapiro–Wilk tests), data were analyzed with non-parametric tests. Friedman’s tests (followed by Wilcoxon *post hoc* test, when applicable) were used to assess differences in pain and taste (sugar and salt) scores. Spearman’s correlations were used to assess relations between the tasks (FAST and taste) and interoception measures (heartbeat task and MAIA), as well associations with pain-related psychological questionnaires (PSS and HADS). In all figures, data were presented as mean ± SD unless specified otherwise. Statistical significance was defined as *P* ≤ 0.05.

## Results

### Participants’ Characteristics

The study sample included 60 volunteers (29 men and 31 women), ranging in age from 18 to 53 with mean ± SD of 23.63 ± 6.31 years. Table 1 depicts the sociodemographic data of the entire sample.

**TABLE 1:** Demographics of the study population (*N* = 60).

**Characteristics**	**Mean ± SD**
Age	23.63 ± 6.31
BMI (kg/m^2^)	22.7 ± 3.5
	Frequency (%)
**Gender**	
Male	29 (48.3%)
Female	31 (51.7%)
**Education**	
High school	40 (66.7%)
Undergraduate	18 (30%)
Graduate	2 (3.3%)
**Marital Status**	
Single	56 (93.3%)
Married	4 (6.7%)

*SD, standard deviation. Data are n (%) or mean (SD).*

### FAST Outcome Measures

**FIGURE 1:**
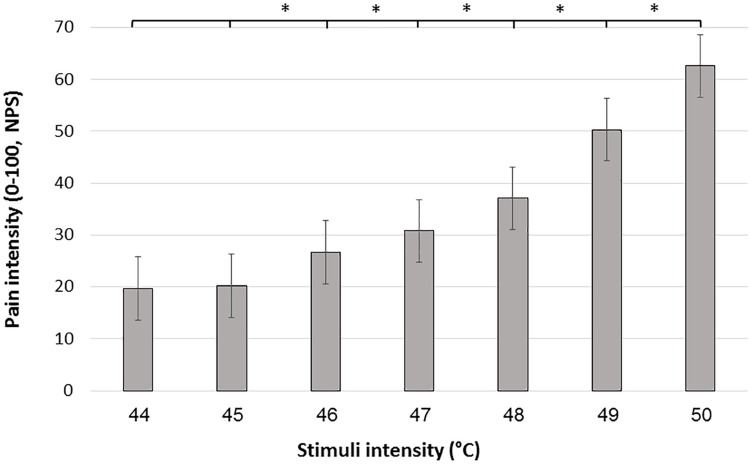
Mean pain scores in response to the 7 stimulus intensities during the FAST paradigm. Black bars represent the average pain scores in response to the seven administrations of each intensity. Error bars represent the standard error of the mean. ^∗^ denotes *P* < 0.05.

Mean pain intensities reported in response to each of the seven stimuli intensities are presented in Figure 1. Group mean ± SD responses ranged from 19.65 ± 17.7 for the lowest stimulus intensity (44°C) to 62.59 ± 23.8 for the highest stimulus intensity (50°C). Mean pain scores significantly differed from each other (Friedman’s test, chi-square 288.83; *P* < 0.001). *Post hoc* Wilcoxon test revealed significant differences between all stimuli intensities (*P* < 0.001) apart from a non-significant difference when comparing between 44°C and 45°C (*P* = 0.216).

Descriptive statistics of the FAST outcomes are described in Table 2. The *R*^2^, ICC, and CoV were widely distributed, suggesting that subjects’ ability to reliably report pain was widely distributed. *R*^2^ had a mean of 0.45 (range = 0.01–0.77), ICC had mean of 0.60 (range = 0.08–0.87), and CoV had a mean of 0.58 (range = 0.05–1.56).

**TABLE 2:** FAST outcomes.

	**R^2^**	**ICC**	**CoV**
Mean (SD)	0.453 (0.16)	0.602 (0.16)	0.577 (0.38)
Median	0.486	0.615	0.487
Minimum	0.010	0.083	0.051
Maximum	0.770	0.873	1.563

*FAST, Focused Analgesia Selection Test; SD, standard deviation; ICC, intraclass correlation coefficient; CoV, coefficient of variation.*

### Taste Task Outcome Measures

**FIGURE 2:**
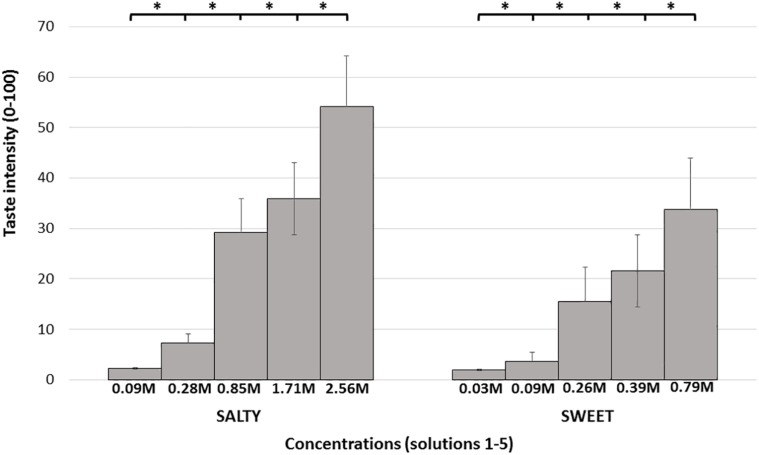
Mean intensity taste scores. Each bar represents the average taste scores in response to the different salty/sweet concentrations. Error bars represent standard errors. Taste concentrations are labeled by concentration (molarity), from lowest to highest concentration. ^∗^ denotes *P* < 0.05.

Mean taste intensity ratings reported in response to each of the five concentrations for both sweet and salty tastes are presented in Figure 2. Group mean ± SD responses ranged from 2.22 (±3.53) and 1.95 (±3.01 SD) for the lowest stimuli intensity up to 54.02 (±25.19) and 33.73 (±25.61) for the highest stimuli intensity (salty and sweet, respectively). Mean taste intensity scores of salt (Friedman’s test, chi-square 231.56, *P* < 0.001) and sugar tastes (Friedman’s test, chi-square 225.15, *P* < 0.001) significantly differed from each other. *Post hoc* Wilcoxon test revealed significant difference between each concentration of both sugar and salt (*P* < 0.001). The taste tests results are described in Table 3. The *R*^2^, ICC, and CoV of taste intensity reports were widely distributed, implying that subjects’ abilities to reliably report the intensity of salty and sweet tastes were widely distributed.

**TABLE 3:** Taste task outcomes.

**SALT**	**R^2^**	**ICC**	**CoV**
Mean (SD)	0.686 (0.14)	0.831 (0.12)	0.475 (0.21)
Median	0.717	0.857	0.442
Minimum	0.340	0.170	0.120
Maximum	0.920	0.960	0.930

**SUGAR**	**R^2^**	**ICC**	**CoV**

Mean (SD)	0.614 (0.18)	0.774 (0.15)	0.496 (0.22)
Median	0.648	0.810	0.470
Minimum	0.030	0.060	0.090
Maximum	0.870	0.960	1.120

*SD, standard deviation; ICC, intraclass correlation coefficient; CoV, coefficient of variation.*

### Interoception Outcome Measures

The raw data of subjective and objective heartbeat perception are described in Table 4. The mean heartbeat perception (interoception) score was 0.65 (±0.23) (range = 0.00–0.98), with a median score of 0.69. This wide range of scores suggests that participants differ in their interoception accuracy, as assessed by the heartbeat detection task. Table 5 depicts the results of the MAIA questionnaire.

**TABLE 4:** Interoception accuracy outcomes.

	**T1 – 25 s**		**T2 – 35 s**		**T3 – 45 s**	
	**Subjective**	**Objective**	**Subjective**	**Objective**	**Subjective**	**Objective**
Mean (SD)	19 (7.1)	27.9 (4.5)	25.3 (10.3)	39.3 (6.0)	33.4 (12.5)	50.9 (7.7)
Median	19	28	26	39	34	51

*Subjective (25, 35, and 45 s) represents mean of the subjective measure for each interval, when subjects are asked to count their heartbeat. Each objective interval represents the mean of number of heartbeats measured through electrocardiogram, for each period of time.*

**TABLE 5:** A summary of the values distribution of the MAIA questionnaire.

**MAIA subscales**	**Mean ± SD**	**Median**	**Min–max**
Noticing	3.38 ± 0.9	3.33	0–5
Not distracting	1.66 ± 0.9	1.50	0–4.5
Not worrying	2.70 ± 1.1	3.0	0.25–5
Attention regulation	3.02 ± 0.8	3.0	1.1–4.7
Emotional awareness	3.64 ± 0.8	3.60	1.80–5
Self-regulation	2.67 ± 0.9	2.57	1–4.43
Trusting	3.81 ± 0.8	4.0	1.67–5

*MAIA, Multidimensional Assessment of Interoceptive Awareness; SD, standard deviation.*

### Cross-Modal Associations

Positive correlation was found between the two taste tasks. Subjects with high salt ICC had high sugar ICC values (Spearman’s *r* = 0.477, *P* < 0.001). That is, the more reliable subjects are in reporting the intensity of salty taste, the more reliable they are in reporting intensity of sweet taste. No significant cross-modal correlations were found between any of the tasks (FAST, taste, and heartbeat task) (*P* > 0.05 for all outcome measures). No significant correlations were found between heartbeat task and MAIA total score (*P* = 0.664) or with any of the MAIA subscales (all outcomes *P* > 0.05).

### Relations Between Pain-Related Psychological Questionnaires and Measures of Reliability

Table 6 depicts psychological characteristics. No significant correlations were found between any of the psychological measures and task results (FAST, taste, and heartbeat).

**TABLE 6:** A summary of the values distribution of the psychological questionnaire.

**Questionnaires**	**Mean ± SD**	**Median**
PSS	18.45 ± 7.15	18.0
HADS-*total*	11.03 ± 6.10	9.0
HADS-*anxiety*	7.03 ± 3.58	7.0
HADS-*depression*	3.95 ± 3.22	3.0

*SD, standard deviation; PSS, perceived stress scale; HADS, Hospital Anxiety and Depression Scale.*

## Discussion

The aim of the current study was to investigate if subjects’ ability to reliably report pain and taste, as reflected by the within-subjects variability of pain and taste reports, relates to interoceptive ability. Our findings revealed no cross-modal relations between the ability to reliably report pain and taste, and interoceptive measures. The only relations found were within the taste modality.

Our results suggest that IAw/ability cannot be generalized across different modalities. Several other studies assessing interoception modalities, such as detection of heartbeat and gastric and respiratory perception, did not find correlations between accuracy of reporting different modalities (Harver et al., 1993; Vaitl, 1996; Werner et al., 2009; Garfinkel et al., 2016; Ferentzi et al., 2017). A recent study directly investigated the relations between six interoception modality tasks, including heartbeat, gastric, pain, bitter perception, proprioception, and balancing ability (Ferentzi et al., 2018). The results indicated that there were no correlations or a common factor between different modalities. As in our case, correlations within measures of the same sensory modality were shown. This suggests that within-subjects’ variability is a characteristic that can be generalized within, but not between modalities. In contrast to our and Ferentzi et al.’s (2018) results, previous studies have shown moderate associations between heartbeat perception task and gastric perception (Whitehead and Drescher, 1980; Herbert et al., 2012; Garfinkel et al., 2016) and between the heartbeat task and the perception of skin conductance (Steptoe and Noll, 1997).

Methodological differences can account for these seemingly contrasting findings. In most studies, methods involved not only different sensory modalities but also different tasks. For example, in Ferentzi et al. (2018), pain was assessed using threshold and tolerance tasks. These two measures assess pain sensitivity and, as such, these measures do not reflect interoceptive ability (if these tests were repeated multiple times, and variability would have been assessed, it could provide a measure of subjects ability to reliably report pain). Some studies showed correlations between heartbeat perception task and pain sensitivity (Herbert et al., 2007; Herbert and Pollatos, 2012; Pollatos et al., 2012; Weiss et al., 2014) while others did not (Werner et al., 2009). Unlike pain sensitivity measures (e.g., pain thresholds and tolerance), the FAST outcomes capture different aspects of pain reporting variability, presumably reflecting, at least partly, pain reporting reliability (which is independent of pain sensitivity). Similarly, a single Visual Analog Scale score of intensity (or unpleasantness) of a taste solution represents the sensitivity of subjects to a given taste (as done by Ferentzi et al., 2018), rather than subject’s ability to reliably report intensity of tastes.

Aligned with previous research findings, in our study, the interoception task was not significantly correlated with the measures assessed by the MAIA questionnaire, suggesting that interoceptive-related tasks might not measure the same constructs as the MAIA. This observation is in line with the theoretical model proposed by Garfinkel et al. (2015). According to the latter, “interoceptive sensibility,” the subjective self-evaluated trait assessed by questionnaires (e.g., MAIA) is not the same construct as “interoception accuracy,” assessed by a task (Calì et al., 2015; Garfinkel et al., 2015).

Our findings of lack of cross-modal correlations support the notion that the ability to reliably report sensations is specific for each sensory system and cannot be generalized across modalities or inferred from one modality to another. Recently, Smith and Lane (2015) proposed that interoception is processed on three hierarchical systems: the “generative” system, processing information from each sensory system (involving somatosensory cortex and posterior insula); the “perceptual” system, where a first “whole-body pattern” is constructed (in the anterior insula); and the “regulatory” system, the final stage where the emotional concepts are created using information from the lower-level processing with higher-level processing (Smith and Lane, 2015). The lack of cross-modal correlations observed in our study is aligned with this model.

Treister et al. (2018) recently demonstrated that, upon training, subjects’ performance in the FAST is improved. The lack of cross-modal correlations found in this study suggests that improving reporting reliability of one modality will probably not affect the reporting reliability of other modalities.

Few limitations deserve consideration. First, the negative results may be a consequence of inadequate statistical power due to the number of participants. However, power calculations supported the recruitment of about 60 subjects, as done in this study. In addition, the fact that those similar negative findings were shown in previous studies supports our results. Second, this study used NRS, which has been related to bias due to the subject’s tendency to cluster their answers near the number labels (Hayes et al., 2013). Nevertheless, Hayes et al. (2013) considered that these scales as sufficient. Third, subjects were not screened to assure baseline taste perception, and even though smoking was restrained before the study, the enrollment of smokers could have biased the results on the taste task.

In summary, our results support the notion that the tendency of subjects to reliably report their sensations cannot be generalized across modalities. Further investigation is ongoing to better understand the clinical relevance of variations in the within-subjects intensity reports of pain and other subjective symptoms.

## Ethics Statement

This study was carried out in accordance with the Declaration of Helsinki, and with the approval of the local Ethical Board. Written informed consent was obtained from each participant before the beginning of the experiment.

## Author Contributions

MA preformed the data collection and supported with the manuscript preparation. RC and RT supported all aspects of the study, from conceptualization and designing, data analyses to manuscript drafting. LH supported the data processing, analyzing, and manuscript preparation.

## Conflict of Interest Statement

The authors declare that the research was conducted in the absence of any commercial or financial relationships that could be construed as a potential conflict of interest.
